# Cross-cultural adaptation of discrimination and vigilance scales in ELSA-Brasil

**DOI:** 10.11606/s1518-8787.2022056004278

**Published:** 2022-11-18

**Authors:** Rosane Harter Griep, Fernanda Esthefane Garrides Oliveira, Odaleia Barbosa de Aguiar, Arlinda B. Moreno, Márcia Guimarães de Mello Alves, Ana Luisa Patrão, Maria de Jesus Mendes da Fonseca, Dóra Chor

**Affiliations:** I Fundação Oswaldo Cruz Instituto Oswaldo Cruz Laboratório de Educação em Ambiente e Saúde Rio de Janeiro RJ Brasil Fundação Oswaldo Cruz. Instituto Oswaldo Cruz. Laboratório de Educação em Ambiente e Saúde. Rio de Janeiro, RJ, Brasil; II Fundação Oswaldo Cruz Escola Nacional de Saúde Pública Sérgio Arouca Programa de Epidemiologia em Saúde Pública Rio de Janeiro RJ Brasil Fundação Oswaldo Cruz. Escola Nacional de Saúde Pública Sérgio Arouca. Programa de Epidemiologia em Saúde Pública. Rio de Janeiro, RJ, Brasil; III Universidade do Estado do Rio de Janeiro Instituto de Nutrição Departamento de Nutrição Aplicada Rio de Janeiro RJ Brasil Universidade do Estado do Rio de Janeiro. Instituto de Nutrição. Departamento de Nutrição Aplicada. Rio de Janeiro, RJ, Brasil; IV Fundação Oswaldo Cruz Escola Nacional de Saúde Pública Sérgio Arouca Departamento de Epidemiologia e Métodos Quantitativos Rio de Janeiro RJ Brasil Fundação Oswaldo Cruz. Escola Nacional de Saúde Pública Sérgio Arouca. Departamento de Epidemiologia e Métodos Quantitativos. Rio de Janeiro, RJ, Brasil; V Universidade Federal Fluminense Instituto de Saúde Coletiva Departamento de Planejamento em Saúde Niterói RJ Brasil Universidade Federal Fluminense. Instituto de Saúde Coletiva. Departamento de Planejamento em Saúde. Niterói, RJ, Brasil; VI Universidade do Porto Faculdade de Psicologia e Ciências da Educação Centro de Psicologia Porto Portugal Universidade do Porto. Faculdade de Psicologia e Ciências da Educação. Centro de Psicologia. Porto, Portugal

**Keywords:** Social Discrimination, classification, Psychometrics, Translating, Reproducibility of Results, Validation Study

## Abstract

**OBJECTIVE:**

To describe the process of cross-cultural adaptation for the use in Brazil of the everyday discrimination scale (EDS) and the heightened vigilance scale (HVS) applied in the Longitudinal Study of Adult Health (ELSA-Brasil).

**METHODS:**

Conceptual, item and semantic equivalence analyses were conducted by a group of four epidemiologists; evaluation of measurement equivalence (factorial analysis of configural, metric and scalar structures, according to sociodemographic characteristics) and reliability. A total of 11,987 participants responded to the discrimination scale, and a subsample of 260 people participated in the test-retest study. In the case of HVS, 8,916 people responded, while 149 individuals did so in the test-retest study.

**RESULTS:**

The scales presented conceptual, item and semantic equivalence pertinent in the Brazilian context, in addition to adequate correspondence of referential/denotative meaning of terms and also of the general/connotative of the items. The confirmatory factor analysis of EDS revealed a unidimensional structure, with residual correlations between two pairs of items, presenting configural and metric invariance among the four subgroups evaluated. Scalar invariance was identified according to sex and age group, but it was not observed for race/color and education. Heightened vigilance showed low loads and high residuals, with inadequate adjustment indicators. For the items of the discrimination scale the weighted kappa coefficient (Kp) ranged from 0.44 to 0.78, and the intraclass correlation coefficient (ICC) was 0.87. For HVS items, the Kp ranged from 0.47 to 0.59 and the ICC was 0.83.

**CONCLUSIONS:**

Although there are correlated items, it was concluded that the EDS is a promising scale to evaluate experiences of perceived discrimination in Brazilian daily life. However, the heightened vigilance scale did not present equivalence of measurement in the current format.

## INTRODUCTION

In recent decades, studies evaluating the association between discrimination and health have increased considerably^1,2^. Its results are part of the documentation of health disparities in the United States (US), due to the relationships established between experiences of discrimination and worse mental and physical health indicators^3,4^. Part of this evidence emerges from studies of interpersonal discrimination, in which experiences of hostility events in everyday life have received greater emphasis^5^, for being a measure of chronic exposure to psychosocial stressors^3,6^.

The perception of everyday discrimination involves unfair and recurrent practices in interpersonal interactions in different contexts and environments, including manifestations of disrespectful treatment, belittlement and offer of worse care or service^6^. The everyday discrimination scale (EDS)^7^ is one of the most widely used instruments to assess racial/ethnic discrimination, especially in the US, but also in European countries, Canada, and South Africa^8^. Originally proposed in the context of the Detroit Area Study, to assess experiences and frequency of self-reported discrimination of racial/ethnic groups and its impact on health^7^, the scale attempts to capture subtler chronic or episodic aspects of interpersonal discrimination^2,4^.

The diffusion of EDS was favored by its brevity and psychometric qualities described in its first decade of use^1,2,7,9^, focused primarily on their performance in different racial/ethnic groups, such as African Americans^9^and Latinos in the US^10^, and among women^11^. These studies suggest good psychometric performance, but recommend that it be evaluated among heterogeneous social groups, which include racial, gender identity and social class diversity. In addition, they question whether the scale should be used to assess the general perception of discrimination, in addition to racial discrimination in which it was more appropriate^12^.

Discrimination-related vigilance has been emphasized as an important component of the association between experiences of discrimination and health events^15^. It is a coping mechanism that is characterized by the physical and mental preparation of the individual, with continuous monitoring of the environment and what happens around it, as well as constant re-adaptation, in order to protect oneself or avoid an experience of discrimination^15,16^. To assess this component of discrimination, the heightened vigilance scale (HVS) was also proposed for the Detroit Area Study to be used in sequence to the EDS, applied to those who responded having experienced previous experiences of discrimination^15^.

The confrontation associated with the HVS has been linked to health problems in the US^15,17^, such as stress^18^. In addition, it was also considered a potential mediator in the theoretical model that relates race/ethnicity and adverse health outcomes^16^. However, the knowledge about its psychometric properties is limited, since the measurement and analysis of the structure of its construct have been little explored^19^.

In order to study the effects of racial discrimination on health, both scales were included in the questionnaire of the third stage (wave 3) of follow-up in the Longitudinal Study of Adult Health (ELSA-Brasil). This article aims to describe the process of cross-cultural adaptation for the use of the EDS and the HVS in Brazil.

## METHODS

### Conceptual, Item and Semantic Equivalences

After authorization of the scales’ authors, we went to the stages of cross-cultural adaptation based on the recommendations of the literature^20^.

Conceptual and item equivalences were evaluated by a group of four epidemiologist researchers with previous experience in the use of scales and/or with the theme of racial discrimination. The process involved an extensive literature review of the use of the instruments, the previous psychometric performance and the relevance of the scales to the Brazilian context.

Semantic equivalence involved four steps: 1) translation of the original instrument in English to Brazilian Portuguese, independently, by two experienced researchers fluent in English. The translators used a standardized form assigning a grade (between 0 and 10) to the degree of difficulty encountered in the translation. The translations generated a consensus version in Portuguese, made by the team of researchers, with the presence of the translators; 2) back-translation of the consensus version in Portuguese, performed by a native translator in English, who recorded comments and evaluated the degree of difficulty in back-translation in notes, also with variation between 0 and 10; 3) comparison of the original version of the scale with that elaborated after back-translation, evaluating the semantic equivalence of the two versions (original and back-translated), in order to ensure the transfer of the meanings of words in both languages. After adjustments and adaptations, corrected versions of the instrument were developed for pre-tests; 4) pre-tests and pilot study of the proposed versions (50 and 18 volunteers, respectively, with similar characteristics to the study population).

### Measurement Equivalence

After applying the scales in wave 3 of ELSA-Brasil, the dimensional structure was evaluated - via exploratory factor analysis (EFA) and confirmatory factor analysis (CFA) – and internal structure adequacy assessment, with examination of invariance of the configural, metric and scalar structures between subgroups of self-reported race/skin color, sex, age group and education.

The ELSA-Brasil is a prospective cohort that, in its baseline (2008–2010), enrolled 15,105 participants aged between 35 and 74 years of both sexes, active and retired employees of six Brazilian educational and research institutions, to monitor chronic health outcomes^21^. Wave 3 of ELSA-Brasil took place between 2017 and 2019, applying face-to-face interviews to 12,636 participants. A total of 11,987 individuals who responded to the discrimination scale participated in the analyses of these; 9,916 reported experiences of discrimination and responded to the HVS.

ELSA-Brasil was approved by the Research Ethics Committee of each of the institutions involved, by the National Research Ethics Commission (Conep 976/2006) and all participants signed an informed consent form.

### Dimensional Structure and Invariance between Subgroups

The existence of previous models on the dimensionality of the discrimination scale^2,22^ guided the psychometric evaluation of this instrument from the CFA. Given the limited knowledge about the psychometric properties of the vigilance scale, EFA was initiated, followed by CFA. KMO index and Bartlett’s sphericity test were used to verify the adequacy of the data and parallel analyses as criteria to verify the retention of factors. The estimation of the parameters was performed by Weighted Least Squares Mean and Variance Adjusted (WLSMV), with implementation of a polychoric matrix^23^. The minimum criterion adopted for the standardized load of the items was 0.50 and loads ≥ 0.70 were considered ideal^24^. In addition, the evaluation of the residual correlations between the items, the modification indices and the values of expected changes of parameters were explored.

To evaluate the adequacy of the model, three indices were considered: the Comparative Fit Index (CFI), the Tucker-Lewis Index (TLI) and the root mean square error of approximation (RMSEA). RMSEA values of less than 0.06 are preferable, but up to 0.08 are acceptable. Its 95% confidence interval (95%CI) was also considered as an additional assessment, with the upper limit not exceeding 0.08. Models with good fit have CFI and TLI of approximately 1: indices ≥ 0.90 are acceptable and ≥ 0.95 are preferable^24^. Convergent validity was evaluated by the average variance extracted (AVE) and internal consistency by the composite reliability (CR), being considered acceptable values when AVE ≥ 0.50 and CR ≥ 0.60^24^.

In the case of day-to-day discrimination, multiple-group CFA was conducted for self-reported race/skin color, gender, age groups, and schooling to assess whether each subgroup has the same structure identified in the CFA without stratification. For comparison of invariance with those who self-declared white, the indigenous (n = 98) and yellow/Asian (n = 308) groups were excluded due to the low proportion among the participants, while the categories of blacks and browns were united, since they presented similar adjustment parameters in the models configured by racial subgroups.

The results of the configural models, by subgroup, with acceptable adjustments, allowed to proceed with the tests for measurement equivalence. This step consists of comparing the subgroups, from three sequential and interdependent models: 1) the reference model: less restricted and allows for evaluation of the configural invariance, that is, the equivalence of the factor structure, if the number of factors and the distribution of items among them are maintained among the subgroups of race/skin color, sex, age group and education; 2) metric invariance: evaluates the equivalence of the pattern of factor loads between subgroups, thereby adding a constraint to the first model: equal factor loads between subgroups; and 3) scalar invariance: evaluates the equivalence of intercepts, that is, whether individuals with the same score in the latent construct would obtain a similar score in the observed variable, regardless of the subgroup of which it is part. This model constrains not only the factor loads, but the variances to be equal between groups^24,25^.

At each stage, we compared the adjustment indices with the indices of the previous model, mainly evaluating the magnitude and direction of the variations in the incremental indices: if the more restrictive model showed a reduction in the CFI ≥ 0.010 complemented by an increase in the RMSEA ≥ 0.015^26^, then the invariance hypothesis was rejected. The chi-square test (χ^2^) for model comparison was used, but interpreted with caution due to its sensitivity to sample size^25^. All analyses of these steps were conducted in the software Mplus 8.12.

### Test-retest Reliability Study

To evaluate the temporal stability of the items and scores, the EDS and HVS were applied twice with an interval between seven and 14 days (mean of ten days) in a random subsample of 260 participants, of which 149 reported experiences of discrimination and also responded to HVS.

Intra-observer reproducibility was assessed by the kappa coefficient with quadratic weighting (Kp), interpreted according to Fleiss’s recommendations^27^. The intraclass correlation coefficient was used to evaluate the reproducibility of the scale scores in the test and retest, and the results were considered satisfactory when they reached minimum values of 0.70^27^. These analyses were conducted in R software, version 4.0.3, with a significance level of 5%.

## RESULTS

The literature review on the topic of discrimination and the debate among researchers specialized in the area showed that there was conceptual and item equivalence, pointing to the relevance of the original instrument in our culture.

Most of the EDS items were considered of little difficulty for translation. Only five items were considered of moderate difficulty and generated some inconsistency among translators or debate among researchers, because they had unusual terms and expressions in our context. All change decisions, described below, were corroborated in the pre-test rounds: (1) *You receive poorer service than other people at restaurants or stores*: in this case, the expression *poorer service* was adapted for the equivalent in Portuguese of “worst quality care”; (2) *You are threatened or harassed*: since we did not find a colloquial term for harassment, we opted for “threatened or harassed/embarrassed”; (3) *You are followed around in stores*: using the expression “be followed” might not represent well the semantic content of the item, since in Brazil some sellers are instructed to follow customers as a sign of greater attention paid to them. As such, we adapted it to “treated suspiciously and is watched in places like stores.” In the evaluation of the translators and in subsequent pretests, there were no difficulties in the translation or interpretation of the HVS items.

The original version of the scales, as well as its final version, are presented in Chart. The discrimination and vigilance scales are formed, respectively, by 10 and six situations/items with answers in the Likert format. For the EDS, each answer was scored from 1 (never) to 6 (almost every day), with a score ranging from six to 60 points; for the HVS it ranged from 1 (never) to 5 (very often), with a total score between six and 30 points. The higher the score, the more frequent the experience of discrimination and vigilance.

**Chart:** Items of the everyday discrimination scale and the heightened vigilance scale, original version and final version in Brazilian Portuguese.

Original English version	Final version in Brazilian Portuguese
Everyday discrimination scale (EDS)	*Escala de discriminação no dia a dia* (EDD)
In your day-to-day life, how often do any of the following things happen to you?	*No seu dia-a-dia, com que frequência as seguintes situações acontecem com o(a) Sr(a)?*
1. You are treated with less courtesy than other people are.	*1. O(a) Sr(a) é tratado(a) com menos gentileza do que as outras pessoas.*
2. You are treated with less respect than other people are.	*2. O(a) Sr(a) é tratado(a) com menos respeito do que as outras pessoas.*
3. You receive poorer service than other people at restaurants or stores.	*3. Em restaurantes e lojas, o(a) Sr(a) recebe um atendimento de pior qualidade do que as outras pessoas.*
4. People act as if they think you are not smart.	*4. As pessoas agem como se o(a) Sr(a) não fosse inteligente.*
5. People act as if they are afraid of you.	*5. As pessoas agem como se tivessem medo do(a) Sr(a).*
6. People act as if they think you are dishonest.	*6. As pessoas agem como se o(a) Sr(a) fosse desonesto(a).*
7. People act as if they’re better than you are.	*7. As pessoas agem como se fossem melhores do que o(a) Sr(a).*
8. You are called names or insulted.	*8. O(a) Sr(a) é xingado(a) ou insultado(a)/ofendido(a).*
9. You are threatened or harassed.	*9. O(a) Sr(a) é ameaçado(a) ou assediado(a)/constrangido(a).*
10. You are followed around in stores.	*10. O(a) Sr(a) é tratado(a) de forma suspeita e é vigiado(a) em lugares como lojas.*
Answer categories: almost every day, at least once a week, a few times a month, a few times a year, less than once a year, never	*Categorias de resposta: quase todo dia, pelo menos uma vez por semana, algumas vezes por mês, algumas vezes por ano, menos de uma vez ao ano, nunca*
Heightened vigilance scale (HVS)	*Escala de vigilância intensificada* (EVI)
In dealing with these day-to-day experiences that you just told me about, how often do you:	*Vamos continuar pensando em como o(a) Sr(a) lida com aquela(s) situação(ões) que relatou anteriormente... No seu dia a dia, com que frequência o(a) Sr(a)…*
1. Think in advance about the kinds of problems you are likely to experience?	*1. ... se prepara com antecedência para os problemas que poderá enfrentar?*
2. Try to prepare for possible insults before leaving home?	*2. ... tenta se preparar para possíveis xingamentos ou insultos ou ofensas antes de sair de casa?*
3. Feel that you always have to be very careful about your appearance to get good service or avoid being harassed?	*3. ... sente que o(a) Sr(a) sempre tem que ser muito cuidadoso(a) em relação à sua aparência para obter um bom serviço ou para evitar ser assediado(a)?*
4. Carefully watch what you say and how you say it?	*4. ... fica cuidadosamente atento (a) ao que fala e como o(a) Sr(a)fala?*
5. Carefully observe what happens around you?	*5. ... observa com cuidado o que acontece no seu entorno?*
6. Try to avoid certain social situations and places?	*6. ... tenta evitar certos eventos sociais e certos lugares?*
Answer categories: very often, fairly often, not too often, hardly ever, never	*Categorias de resposta: com muita frequência, com frequência, com pouco frequência, quase nunca, nunca*

### Dimensional Structure and Invariance Between Subgroups

The study participants were mostly women (56%), in the age range of 40 to 59 years (53.8%), with complete higher education (58%) and self-declared as white (52%). Similar characteristics were observed among participants in the subsample of the test-retest reliability study.

In the CFA for the discrimination scale (Table 1), the initial model, without correlations between the items, presented loads between 0.527 (item 5) and 0.865 (item 2), with adjustment indices far from the recommended limits: CFI = 0.931; TLI = 0.911; RMSEA = 0.122. After inserting the residual correlations between items 1 and 2, the adjustment indices improved. The result with a single factor showed a reasonable adjustment with improvement of the indicators after an additional correlation: between items 8 and 9 of the scale: CFI = 0.980; TLI = 0.973; RMSEA = 0.067. The AVE (0.402) was lower than the acceptable limit (≥ 0.500), but the CR (0.869) reached a preferable value (≥ 0.600). This CFA model with two correlations between items served as the basis for the models configured by subgroup of sociodemographic characteristics and the analysis of invariance in the EDS.

**Table 1:** Confirmatory factor analysis and adjustment parameters for the everyday discrimination scale (EDS), ELSA-Brasil, 2017–2019, (n = 11,987).

Scale items, residual correlations and adjustment indices	CFA (uncorrelated)		CFA (correlation 1 and 2)		Final CFA	
		
	CF	R	CF	R	CF	R
**Everyday discrimination scale**						
1. Treated with less courtesy	0.825	0.319	0.671	0.550	0.676	0.542
2. Treated with less respect	0.865	0.252	0.724	0.476	0.731	0.466
3. You receive poorer service	0.648	0.580	0.673	0.547	0.677	0.542
4. People act as if they think you are not smart	0.726	0.473	0.757	0.426	0.762	0.420
5. People act as if they are afraid of you	0.527	0.723	0.546	0.702	0.549	0.699
6. People act as if they’re better than you are	0.714	0.492	0.734	0.461	0.740	0.453
7. People act as if they think you are dishonest	0.710	0.496	0.750	0.437	0.754	0.431
8. You are called names or insulted	0.678	0.540	0.705	0.503	0.667	0.555
9.You are threatened or harassed/embarrassed	0.678	0.541	0.701	0.508	0.657	0.568
10. You are treated suspiciously and watched in places such as stores	0.637	0.594	0.656	0.569	0.661	0.564
Residual correlation - items 1 and 2	-	-	0.605	-	0.599	-
Residual correlation - items 8 and 9	-	-	-	-	0.329	-
**Adjustment indices**						
Tucker-Lewis comparative adjustment index (IFC/TLI)	0.931/0.911		0.973/0.964		0.980/0.973	
RMSEA (95%CI)	0.122 (0.120–0.125)		0.078 (0.075–0.810)		0.067 (0.065–0.700)	
Average variance extracted (AVE)	-		-		0.402	
Composite reliability (CR)	-		-		0.869	

CFA: confirmatory factor analysis; CF: standardized factor loadings; R: residuals; RMSEA: root mean square error of approximation; 95%CI: 95% confidence interval.

For the evaluation of the HVS, we proceeded to the EFA, initially, by the method of extraction of the main axes, oblique rotation. The KMO index for sample adequacy was 0.75, considered good, indicating that the matrix was factorable. Bartlett’s sphericity test with significance levels p < 0.00 was considered adequate, indicating that the correlational matrix was not an identity matrix. Parallel analyses indicated the retention of two factors. Exploratory factor analysis with one factor showed very low loads for items 5 and 6: 0.495 and 0.363, respectively. This configural model also did not present good adjustment indicators: CFI = 0.918; TLI = 0.864; RMSEA = 0.138. The results of EFA with two factors improved these indicators, but showed cross-loads in items 3 and 6, as well as residues with high values. The CFA with single factor solution presented acceptable results after insertion of residual correlations between items 1 and 2 and between items 4 and 5 of the scale: CFI = 0.991; TLI = 0.980; RMSEA = 0.053. However, items 5 and 6 continued with loads less than 0.500. The composite reliability result for this model (0.668) was satisfactory, but the convergent validity (0.258) was lower than recommended (Table 2). With low loads and high residuals in the confirmatory factor analysis of the HVS, we chose not to proceed with the models configured by subgroup and with the tests for equivalence of measurement of this scale.

**Table 2:** Exploratory factor analysis, confirmatory factor analysis and adjustment parameters for the heightened vigilance scale (HVS), ELSA-Brasil, 2017–2019, (n = 8,916).

Scale items, residual correlations and adjustment indices	EFA (1 factor)		EFA (2 factors)			CFA		Final CFA	
			
	CF	R	CF1	CF2	R	CF	R	CF	R
**Heightened vigilance scale**									
1. You prepare in advance for the problems you may face	0.698	0.512	0.535	0.215	0.563	0.531	0.718	0.564	0.682
2. You try to prepare for possible name-calling or insults or offenses before leaving the house	0.771	0.406	0.991	0.003	0.020	0.596	0.644	0.634	0.599
3. You feel that you always have to be very careful about your appearance	0.577	0.667	0.280	0.382	0.679	0.627	0.606	0.653	0.574
4. You are very cautious to what you say and how you say it	0.641	0.589	0.002	0.824	0.322	0.716	0.487	0.641	0.589
5. You watch carefully what happens in your surroundings	0.495	0.755	0.000	0.559	0.688	0.534	0.715	0.434	0.812
6. You try to avoid certain social events and certain places	0.363	0.868	0.132	0.287	0.866	0.387	0.850	0.395	0.844
Residual correlation - items 1 and 2	-	-	-	-	-	0.456	-	0.422	-
Residual correlation - items 4 and 5	-	-	-	-	-	-	-	0.265	-
**Adjustment indices**									
Tucker-Lewis comparative adjustment index (IFC/TLI)	0.918 / 0.864		0.996 / 0.986			0.973 / 0.949		0.991 / 0.980	
RMSEA (95%CI)	0.138 (0.132–0.144)		0.044 (0.035–0.053)			0.085 (0.078–0.091)		0.053 (0.046–0.060)	
Correlation of dimensions	-		0.456			-		-	
Average variance extracted (AVE)	-		-			-		0.258	
Composite reliability (CR)	-		-			-		0.668	

EFA: exploratory factor analysis; CFA: confirmatory factor analysis; CF: standardized factor loadings; R: residuals; RMSEA: root mean square error of approximation; 95%CI: 95% confidence interval.

The evaluation of measurement equivalence for EDS showed the indicators CFI (≥ 0.95) and RMSEA (≤ 0.080) with acceptable adjustments for configural invariance in the comparisons of the four groups. In the metric invariance, these adjustment indicators improved in all group comparisons, i.e., the RMSEA was significantly reduced, without overlapping the 95%CI with the estimate of the previous model, and there was an increase in the CFI that was greater than 0.990 in all cases. The scalar invariance model indicated constancy only for comparisons in sex subgroups. For comparison between age groups, the reduction in CFI was borderline, going from 0.992 in the metric invariance model to 0.981 in the scalar model (Δ of -0.011), while the increase in RMSEA was tolerable (Δ of 0.007). Scalar invariance was not reached for comparisons in the race/skin color and education subgroups. In both cases, the increase in RMSEA was greater than 0.015 (0.023 and 0.016, respectively) and the reduction in CFI was greater than 0.010 (-0.024 and -0.018, respectively) (Table 3).

**Table 3:** Comparison of the invariance between subgroups of socioeconomic characteristics for configural, metric and scalar equivalences of the everyday discrimination scale. ELSA-Brasil, 2017–2019 (n = 11,987).

Measurement equivalence	Parameters and quality indices for the adjustment of models							Comparison of models (p)	
	
	χ^2^ (gl)	p	Free parameters	RMSEA (95%CI)	∆_RMSEA_	CFI	∆_CFI_	Metric and configural	Scalar and metric
Race/skin color									
Configural invariance	1,731.539 (66)	< 0.0001	124	0.066 (0.064–0.069)	0.001	0.981	0.001	-	-
Metric invariance	731.981 (76)	< 0.0001	114	0.039 (0.036–0.041)	-0.027	0.993	0.012	0.0348	-
Scalar invariance	2,878.628 (126)	< 0.0001	64	0.062 (0.060–0.064)	0.023	0.969	-0.024	-	< 0.0001
Sex									
Configural invariance	1,875.612 (66)	< 0.0001	124	0.068 (0.065–0.070)	0.003	0.980	0.000	-	-
Metric invariance	876.957 (76)	< 0.0001	114	0.042 (0.039–0.044)	-0.026	0.991	0.011	< 0.0001	-
Scalar invariance	1,052.410 (126)	< 0.0001	64	0.035 (0.033–0.037)	-0.007	0.990	-0.001	-	< 0.0001
Age groups									
Configural invariance	1,911.151 (66)	< 0.0001	124	0.068 (0.066–0.071)	0.003	0.980	0.000	-	-
Metric invariance	825.650 (76)	< 0.0001	114	0.041 (0.038–0.043)	-0.027	0.992	0.012	0.0073	-
Scalar invariance	1,876.840 (126)	< 0.0001	64	0.048 (0.046–0.050)	0.007	0.981	-0.011	-	< 0.0001
Schooling level									
Configural invariance	1,856.748 (66)	< 0.0001	124	0.068 (0.065–0.070)	0.003	0.981	0.001	-	-
Metric invariance	855.991 (65)	< 0.0001	114	0.042 (0.039–0.044)	-0.026	0.992	0.011	< 0.0001	-
Scalar invariance	2,604.448 (126)	< 0.0001	64	0.058 (0.056–0.060)	0.016	0.974	-0.018	-	< 0.0001

gl: degrees of freedom; RMSEA: root mean square error of approximation; 95%CI: 95% confidence interval; CFI: comparative fit index.

### Test-retest Reliability Study

For the EDS, the test and retest scores ranged respectively from 10 to 48 (mean = 18.07) and from 10 to 51 (mean = 17.05). For HVS, it ranged from seven to 29 (mean = 19.78) in the test and from seven to 30 in the retest (mean = 18.92). The Kp in the EDS ranged from 0.39 (item 9) to 0.78 (item 10), while for the HVS it ranged from 0.47 (item 3) and 0.56 (item 4). The ICC ranged from 0.87 (95%CI: 0.83–0.90) for EDS and 0.83 (95%CI: 0.76–0.88) for HVS (Table 4).

**Table 4:** Test-retest reliability of the everyday discrimination scale (EDS) and the heightened vigilance scale (HVS), ELSA-Brasil, 2017–2019.

	Weighted kappa (95%CI)	Coefficient of intraclass correlation
Everyday discrimination scale (n = 260)		
1. Treated with less courtesy	0.59 (0.49–0.69)	0.87 (95%CI: 0.83–0.90)
2. Treated with less respect	0.58 (0.48–0.68)	
3. You receive poorer service	0.58 (0.45–0.72)	
4. People act as if they think you are not smart	0.66 (0.56–0.76)	
5. People act as if they are afraid of you	0.63 (0.51–0.76)	
6. People act like they’re better than you	0.65 (0.48–0.81)	
7. People act as if they think you are dishonest	0.62 (0.52–0.71)	
8. You are called names or insulted/offended	0.46 (0.32–0.60)	
9. You are threatened or harassed / embarrassed	0.39 (0.20–0.57)	
10. You treated suspiciously and is watched in places such as stores	0.78 (0.68–0.88)	
Heightened vigilance scale (n = 149)		
1. You prepare in advance for the problems you may face	0.50 (0.36–0.63)	0.83 (95%CI: 0.76–0.88)
2. You try to prepare for possible name-calling or insults or offenses before leaving the house	0.51 (0.37–0.65)	
3. You feel that you always have to be very careful about your appearance	0.47 (0.32–0.61)	
4. You are very cautious to what you say and how you say it	0.56 (0.42–0.71)	
5. You watch carefully what happens in your surroundings	0.53 (0.37–0.70)	
6. You try to avoid certain social events and certain places	0.51 (0.38–0.65)	

95%CI: 95% confidence interval.

## DISCUSSION

Following the steps recommended by the literature^20^ regarding cross-cultural adaptation, the results showed that the Brazilian version of the EDS presents acceptable cross-cultural adaptation, which allows its future use in epidemiological studies. However, our analyses do not corroborate the use of the discrimination-related HVS in the current format.

Regarding the EDS, our analysis supported the unidimensionality of the scale, similar to other studies^2,22^, and was consistent with previous research on local dependence or high correlation between items 1 and 2 and between items 8 and 9^13,22,28^. A psychometric study that also qualitatively explored the scale indicated that the correlation between items 1 and 2 may be due to redundancy, since these items were seen as having similar meaning by the respondents^28^, although this was not captured in the pre-tests carried out within the framework of ELSA-Brasil. On the other hand, the correlation between items 8 and 9 can be explained by the nature of these experiences, related to acute forms of discrimination, directly addressed and more evident, and distancing themselves from the subtle or chronic nature of the experiences for which the EDS was proposed^22^.

Taking into account these assessments, a summary version of the five-item discrimination scale (α = 0.77) was developed for the Chicago Community Adult Health Study^29^ and it contemplates changes in items that we identified as correlated: the first two items of the scale were joined (*treated with less courtesy/respect than other people*) and item 8 was removed, keeping only item 9, plus the term “embarrassed”. Items 6, 7 and 10 were not included in the short version. We therefore encourage future research in Brazil and other Portuguese-speaking countries to evaluate the appropriateness of modified summary versions of the scale.

The models used to evaluate the equivalence of measurement between groups in the discrimination scale indicated metric invariance in all cases, providing evidence that respondents use the scale in a similar way between subgroups, so the differences between values can be compared^24^. However, scalar invariance was not achieved for comparisons between racial groups and groups of different schooling levels. These results confirm the conclusions of more recent studies on measurement invariance in EDS, which reported, when considering general discrimination without attributing motivation, lack of equivalence (or non-invariance) between racial groups and based on schooling^12,13^. These studies, however, differed regarding the invariance between other subgroups: while one indicated that only comparisons between age groups of the estimates of the discrimination scale can be considered significant^13^, the other suggested that comparisons between men and women are appropriate^12^. It should be noted, however, that the change in CFI values adopted in these studies, with reductions ≥ 0.002, were considered an indication of non-invariance between groups. This cut-off point was more conservative than the one adopted in our analyses.

Regarding the HVS, unacceptable indicators were observed and, as far as it was possible to evaluate, few studies evaluated the psychometric properties of this scale, which limits comparisons. Similar challenges related to scale dimensionality have been reported in other studies. For example, a study in the USA indicated two dimensions in exploratory and confirmatory factor analyses: one related to what the authors termed *preparation* (1 to 3) and another related to what they called *caution* (from item 4 to 6)^19^. The same study, using a single-factor model, found unsatisfactory adjustment (CFI = 0.94 and RMSEA = 0.12) and chose to follow the analysis with a two-factor model with improvement in indicators (CFI = 0.98 and RMSEA = 0.07). Additional information on the scale structure comes from the study that originally published the HVS and reported principal component analysis, with one component (eigenvalue = 3.42) accounting for 57% of the standardized variance (loads > 0.69)^15^.

These aspects have also motivated the revision of this scale, and a summarized version with four items (α = 0.72) was applied in the Chicago Community Adult Health Study^30^. This version removes two items that were involved in residual correlations in our analysis: items 1 and 5. Another study used an even more summarized version of the HVS with removal of item 4 (α = 0.66)^16^ and LaVeist et al.^17^ used the original version, with the removal of item 6 (α = 0.69).

Although the abbreviated HVS is used in research on its association with health outcomes, additional psychometric assessments are needed in different contexts to elucidate whether the scale can be used in the population and in the comparison between subgroups. We found only one study that evaluated the measurement invariance of HVS, in which comparisons between transgender and cisgender and between cisgender subgroups could be performed, since the scale works equivalently between these groups. On the other hand, comparisons between transgender subgroups required caution, since partial metric invariance and partial scalar invariance were found^19^.

The present study shows as its strengths the quality of the data collection process, as well as a very broad appreciation of the assessments that make up the stages of cross-cultural adaptation in a comprehensive sample in the country and with different sociodemographic characteristics. However, the composition of the population of ELSA-Brasil – younger and older adults, employed or retired, and with higher education than the average of the Brazilian population – limits its representativeness.

Finally, we highlight that the EDS obtained acceptable psychometric results for use in ELSA-Brasil and similar populations. It was not possible to identify acceptable psychometric properties for the vigilance scale. However, given the importance of the theme in epidemiological studies in the Brazilian reality, it is advisable that the most recently proposed summary versions be used in new studies and evaluated on their relevance in our context.
