# Supplementary material for: Cross-cultural adaptation of discrimination and vigilance scales in ELSA-Brasil
Source: Rev Saude Publica. 2022 Nov 18;56:110. doi: 10.11606/s1518-8787.2022056004278 (PMC9749726; doi:10.11606/s1518-8787.2022056004278)
Supplement: Translation

# Translation: Adaptação transcultural de escalas de discriminação e vigilância no ELSA-Brasil

**Authors:** Rosane Harter Griep, Fernanda Esthefane Garrides Oliveira, Odaleia Barbosa de Aguiar, Arlinda B. Moreno, Márcia Guimarães de Mello Alves, Ana Luisa Patrão, Maria de Jesus Mendes da Fonseca, Dóra Chor

**Affiliations:** I Brasil Fundação Oswaldo Cruz. Instituto Oswaldo Cruz. Laboratório de Educação em Ambiente e Saúde. Rio de Janeiro, RJ, Brasil; II Brasil Fundação Oswaldo Cruz. Escola Nacional de Saúde Pública Sérgio Arouca. Programa de Epidemiologia em Saúde Pública. Rio de Janeiro, RJ, Brasil; III Brasil Universidade do Estado do Rio de Janeiro. Instituto de Nutrição. Departamento de Nutrição Aplicada. Rio de Janeiro, RJ, Brasil; IV Brasil Fundação Oswaldo Cruz. Escola Nacional de Saúde Pública Sérgio Arouca. Departamento de Epidemiologia e Métodos Quantitativos. Rio de Janeiro, RJ, Brasil; V Brasil Universidade Federal Fluminense. Instituto de Saúde Coletiva. Departamento de Planejamento em Saúde. Niterói, RJ, Brasil; VI Portugal Universidade do Porto. Faculdade de Psicologia e Ciências da Educação. Centro de Psicologia. Porto, Portugal

**Keywords:** Discriminação Social, classificação, Psicometria, Tradução, Reprodutibilidade dos Testes, Estudo de Validação

## Abstract

**OBJETIVO:**

Descrever o processo de adaptação transcultural para o uso no Brasil das escalas de discriminação no dia a dia (EDD) e vigilância intensificada (EVI), aplicadas no Estudo Longitudinal de Saúde do Adulto (ELSA-Brasil).

**MÉTODOS:**

Foram realizadas análises das equivalências conceitual, de itens e semântica conduzidas por um grupo de quatro epidemiologistas; avaliação da equivalência de mensuração (análise fatorial das estruturas configural, métrica e escalares, segundo características sociodemográficas) e a confiabilidade. Responderam à escala de discriminação 11.987 participantes e uma subamostra de 260 pessoas participaram do estudo teste-reteste. No caso da EVI, 8.916 pessoas responderam e 149 indivíduos no estudo teste-reteste.

**RESULTADOS:**

As escalas apresentaram equivalências conceitual, de itens e semântica pertinentes no contexto brasileiro, além de adequada correspondência de significado referencial/denotativa de termos e também da geral/conotativa dos itens. A análise fatorial confirmatória da EDD revelou estrutura unidimensional, com correlações residuais entre dois pares de itens, apresentando invariância configural e métrica entre os quatro subgrupos avaliados. Identificou-se invariância escalar segundo sexo e faixa etária, mas não foi observada para recortes de raça/cor e escolaridade. A vigilância intensificada apresentou cargas baixas e resíduos altos, com indicadores de ajuste inadequados. Para os itens da escala de discriminação o coeficiente de concordância kappa ponderado (Kp) variou de 0,44 a 0,78, e o coeficiente de correlação intraclasse (CCI) foi 0,87. Para os itens da EVI, o Kp variou de 0,47 a 0,59 e o CCI foi 0,83.

**CONCLUSÕES:**

Embora haja itens correlacionados, concluiu-se que a EDD é uma escala promissora para avaliar experiências de discriminação percebidas no cotidiano brasileiro. Entretanto, a EVI não apresentou equivalência de mensuração no formato atual.

## INTRODUÇÃO

Nas últimas décadas, estudos que avaliaram a associação entre discriminação e saúde aumentaram consideravelmente^1,2^. Seus resultados integram parte da documentação de disparidades em saúde nos Estados Unidos (EUA), devido às relações estabelecidas entre experiências de discriminação e piores indicadores de saúde mental e física^3,4^. Parte dessas evidências emerge dos estudos de discriminação interpessoal, na qual experiências de eventos de hostilidade na vida cotidiana receberam maior ênfase^5^, por ser uma medida de exposição crônica a estressores psicossociais^3,6^.

A percepção de discriminação cotidiana envolve práticas injustas e recorrentes nas interações interpessoais em diferentes contextos e ambientes, incluindo manifestações de tratamento desrespeitoso, menosprezo e oferta de pior atendimento ou serviço^6^. A *everyday discrimination scale* (escala de discriminação no dia a dia – EDD)^7^ está entre os instrumentos mais utilizados para avaliar a discriminação racial/étnica, sobretudo nos EUA, mas também em países europeus, no Canadá e na África do Sul^8^. Originalmente proposta para o contexto do *Detroit Area Study*, para avaliar experiências e frequência de discriminação autorreferida de grupos raciais/étnicos e seu impacto na saúde^7^, a escala tenta capturar aspectos crônicos ou episódicos mais sutis da discriminação interpessoal^2,4^.

A difusão da EDD foi favorecida por sua brevidade e qualidades psicométricas descritas em sua primeira década de uso^1,2,7,9^, concentradas prioritariamente em seu desempenho em diferentes grupos raciais/étnicos, como afro-americanos^9^e latinos nos EUA^10^, e entre mulheres^11^. Esses estudos sugerem bom desempenho psicométrico, mas recomendam que seja avaliado entre grupos sociais heterogêneos, que incluam diversidade racial, de identidade de gênero e classe social. Além disso, questionam se a escala deveria ser usada para avaliar a percepção geral de discriminação, além da discriminação racial em que se mostrou mais adequada^12^.

A vigilância relacionada à discriminação tem sido enfatizada como um componente importante da associação entre experiências de discriminação e eventos de saúde^15^. Trata-se de um mecanismo de enfrentamento que se caracteriza pela preparação física e mental do indivíduo, com monitoramento contínuo do ambiente e do que acontece ao redor, e constante readaptação, a fim de se proteger ou evitar uma experiência de discriminação^15,16^. Para avaliar esse componente da discriminação, a *heightened vigilance scale* (escala de vigilância intensificada – EVI) foi proposta também para o *Detroit Area Study* para ser utilizada em sequência à EDD, aplicada àqueles que responderam terem vivenciado experiências prévias de discriminação^15^.

O enfrentamento avaliado pela EVI foi associado a problemas de saúde nos EUA^15,17^, como o estresse^18^. Além disso, foi considerado também um mediador potencial no modelo teórico que relaciona raça/etnia e desfechos adversos de saúde^16^. Entretanto, o conhecimento sobre suas propriedades psicométricas é limitado, já que a mensuração e a análise da estrutura de seu constructo ainda foram pouco exploradas^19^.

Com o intuito de estudar os efeitos da discriminação racial sobre a saúde, ambas as escalas foram incluídas no questionário da terceira etapa (onda 3) de seguimento do Estudo Longitudinal de Saúde do Adulto (ELSA-Brasil). O presente artigo pretende descrever o processo de adaptação transcultural para o uso no Brasil das escalas EDD e EVI.

## MÉTODOS

### Equivalências Conceitual, de Itens e Semântica

Após a autorização dos autores das escalas, partiu-se para as etapas da adaptação transcultural com base nas recomendações da literatura^20^.

As equivalências conceitual e de itens foram avaliadas por um grupo de quatro pesquisadores epidemiologistas com experiência prévia no uso de escalas e/ou com tema da discriminação racial. O processo envolveu ampla revisão bibliográfica do uso dos instrumentos, do desempenho psicométrico prévio e da pertinência das escalas para o contexto brasileiro.

A equivalência semântica envolveu quatro etapas: 1) tradução do instrumento original em inglês para a língua portuguesa de variante brasileira, de forma independente, por dois pesquisadores experientes e fluentes em inglês. Os tradutores utilizaram um formulário padronizado atribuindo uma nota (entre 0 e 10) ao grau de dificuldade encontrada na tradução. As traduções geraram uma versão de consenso em português, feita pela equipe de pesquisadores, com a presença dos tradutores; 2) retrotradução da versão de consenso em português, realizada por um tradutor nativo na língua inglesa, que registrou comentários e avaliou o grau de dificuldade na retrotradução em notas, também com variação entre zero e dez; 3) comparação da versão original da escala com aquela elaborada após a retrotradução, avaliando-se a equivalência semântica das duas versões (original e retrotraduzida), com o intuito de garantir a transferência dos significados das palavras nos dois idiomas. Após ajustes e adaptações, versões corrigidas do instrumento foram elaboradas para a realização de pré-testes; 4) pré-testes e estudo piloto das versões propostas (50 e 18 voluntários, respectivamente, com características semelhantes à população de estudo).

### Equivalência de Mensuração

Após a aplicação das escalas na onda 3 do ELSA-Brasil procedeu-se a avaliação da estrutura dimensional – via análise fatorial exploratória (AFE) e análise fatorial confirmatória (AFC) – e avaliação de adequação da estrutura interna, com exame de invariância das estruturas configural, métrica e escalar entre subgrupos de raça/cor da pele autorreferida, sexo, faixa etária e escolaridade.

O ELSA-Brasil é uma coorte prospectiva que, na sua linha de base (2008–2010), arrolou 15.105 participantes com idade entre 35 e 74 anos de ambos os sexos, servidores ativos e aposentados de seis instituições brasileiras de ensino e pesquisa, para acompanhamento de desfechos crônicos de saúde^21^. A onda 3 do ELSA-Brasil ocorreu entre 2017 e 2019, aplicando entrevistas face a face a 12.636 participantes. Participaram das análises 11.987 indivíduos que responderam à escala de discriminação, desses, 9.916 relataram experiências de discriminação e responderam à EVI.

O ELSA-Brasil foi aprovado pelo Comitê de Ética em Pesquisa de cada uma das instituições envolvidas, pela Comissão Nacional de Ética em Pesquisa (CONEP 976/2006) e todos os participantes assinaram termo de consentimento livre e esclarecido.

### Estrutura Dimensional e Invariância entre Subgrupos

A existência de modelos prévios sobre a dimensionalidade da escala de discriminação^2,22^ guiou a avaliação psicométrica desse instrumento a partir da AFC. Dado o conhecimento limitado sobre as propriedades psicométricas da escala de vigilância, a AFE foi iniciada, seguida pela AFC. Os testes de índice de KMO e de esfericidade de Bartlett foram utilizados na verificação da adequação dos dados e análises paralelas como critério para verificar a retenção de fatores. A estimação dos parâmetros foi realizada por *Weighted Least Squares Mean and Variance Adjusted* (WLSMV), com implementação de uma matriz policórica^23^. O critério mínimo adotado para a carga padronizada dos itens foi de 0,50 e cargas ≥ 0,70 foram consideradas ideais^24^. Além disso, a avaliação das correlações residuais entre os itens, os índices de modificação e os valores de mudanças esperadas de parâmetros foram explorados.

Para avaliar a adequação do modelo, três índices foram considerados: o de ajuste comparativo (CFI – *Comparative Fit Index*), o de Tucker-Lewis (TLI – *Tucker Lewis Index*) e a raiz do erro quadrático médio de aproximação (RMSEA – *root mean square error of approximation*). Valores de RMSEA inferiores a 0,06 são preferíveis, mas até 0,08 são aceitáveis. Considerou-se ainda seu intervalo de 95% de confiança (IC95%) como avaliação adicional, com o limite superior não ultrapassando 0,08. Modelos com bom ajuste têm CFI e TLI aproximadamente de 1 (um): índices ≥ 0,90 são aceitáveis e ≥ 0,95 são preferíveis^24^. A validade convergente foi avaliada pela variância média extraída (VME) e a consistência interna pela confiabilidade composta (CC), sendo considerados valores aceitáveis quando VME ≥ 0,50 e CC ≥ 0,60^24^.

No caso da discriminação no dia a dia, foi conduzida AFC de múltiplos grupos para raça/cor da pele autorreferida, sexo, faixas etárias e escolaridade para avaliarmos se em cada subgrupo se configura a mesma estrutura identificada na AFC sem estratificação. Para comparação de invariância com brancos, indígenas (n = 98) e amarelos (n = 308) foram excluídos devido à baixa proporção entre os participantes, enquanto as categorias de pretos e pardos foram unidas, pois apresentaram parâmetros de ajuste semelhantes nos modelos configurais por subgrupos raciais.

Os resultados dos modelos configurais, por subgrupo, com ajustes aceitáveis permitiram prosseguir com os testes para equivalência de mensuração. Essa etapa consiste em comparar os subgrupos, a partir de três modelos sequenciais e interdependentes: 1) *o modelo de referência*: menos restrito e possibilita avaliar a invariância configural, ou seja, a equivalência da estrutura fatorial, se o número de fatores e a distribuição dos itens entre eles se mantêm entre os subgrupos de raça/cor da pele, sexo, faixa etária e escolaridade; 2) *invariância métrica*: avalia a equivalência do padrão das cargas fatoriais entre os subgrupos, adicionando dessa forma uma restrição ao primeiro modelo: cargas fatoriais iguais entre subgrupos; e 3) *invariância escalar*: avalia a equivalência de interceptos, isso é, se indivíduos com escore igual no construto latente obteriam escore semelhante na variável observada, independentemente do subgrupo do qual faz parte. Esse modelo restringe não apenas as cargas fatoriais, mas as variâncias para serem iguais entre grupos^24,25^.

Comparamos em cada etapa os índices de ajuste com os índices do modelo anterior, avaliando principalmente a magnitude e direção das variações nos índices incrementais: se o modelo mais restritivo apresentou redução no CFI ≥ 0,010 complementado por aumento no RMSEA ≥ 0,015^26^, então a hipótese de invariância foi rejeitada. O teste de qui-quadrado (χ^2^) para comparação de modelos foi utilizado, mas interpretado com cautela, devido sua sensibilidade ao tamanho amostral^25^. Todas as análises dessas etapas foram conduzidas no *software* Mplus 8.12.

### Estudo de Confiabilidade Teste-reteste

Para avaliar a estabilidade temporal dos itens e dos escores, as escalas EDD e EVI foram aplicadas duas vezes com intervalo entre sete e 14 dias (média de 10 dias) em subamostra aleatória de 260 participantes, dos quais, 149 relataram experiências de discriminação e responderam também à EVI.

A reprodutibilidade intraobservador foi avaliada por meio da concordância do kappa com ponderação quadrática (Kp), interpretado de acordo com recomendações de Fleiss^27^. O coeficiente de correlação intraclasse foi utilizado para avaliar a reprodutibilidade dos escores das escalas no teste e no reteste, e os resultados foram considerados satisfatórios quando alcançaram valores mínimos de 0,70^27^. Essas análises foram conduzidas no software R, versão 4.0.3, com nível de significância de 5%.

## RESULTADOS

A revisão bibliográfica sobre o tema da discriminação e o debate entre os pesquisadores especializados na área mostraram que havia equivalência conceitual e de itens, apontando para a pertinência do instrumento original na nossa cultura.

A maioria dos itens da EDD foi considerada de pouca dificuldade para a tradução. Apenas cinco itens foram considerados de dificuldade moderada e geraram alguma inconsistência entre os tradutores ou debate entre os pesquisadores por possuírem termos e expressões incomuns em nosso contexto. Todas as decisões de mudança, descritas a seguir, foram corroboradas nas rodadas de pré-teste: (1) *You receive poorer service than other people at restaurants or stores*: neste caso, a expressão *poorer service* foi adaptada para “atendimento de pior qualidade”; (2) *You are threatened or harassed*: já que não encontramos um termo coloquial para assédio, optamos por “ameaçado(a) ou assediado(a)/constrangido(a)”; (3) *You are followed around in stores*: utilizar a expressão “ser seguido” poderia não representar bem o conteúdo semântico do item, já que no Brasil alguns vendedores são instruídos a seguir clientes como sinal de maior atenção dispensada a eles. Então, adaptamos para “tratado(a) de forma suspeita e é vigiado(a) em lugares como lojas”. Na avaliação dos tradutores e em pré-testes subsequentes, não houve dificuldades na tradução ou interpretação dos itens da EVI.

A versão original das escalas, bem como sua versão final estão apresentadas no Quadro. As escalas de discriminação e de vigilância são formadas, respectivamente, por 10 e seis situações/itens com respostas em formato *Likert*. Para a EDD, cada resposta foi pontuada de 1 (nunca) a 6 (quase todos os dias), com escore variando entre seis e 60 pontos; para a EVI variou de 1 (nunca) a 5 (com muita frequência), com escore total entre seis e 30 pontos. Quanto maior a pontuação, mais frequente é a experiência de discriminação e a vigilância.

**Quadro:** Itens da escala de discriminação no dia a dia e escala de vigilância intensificada, versão original e versão final em português do Brasil.

Versão original em inglês	Versão final em português do Brasil
*Everyday discrimination scale* (EDS)	Escala de discriminação no dia a dia (EDD)
*In your day-to-day life, how often do any of the following things happen to you?*	No seu dia-a-dia, com que frequência as seguintes situações acontecem com o(a) Sr(a)?
*1. You are treated with less courtesy than other people are.*	1. O(a) Sr(a) é tratado(a) com menos gentileza do que as outras pessoas.
*2. You are treated with less respect than other people are.*	2. O(a) Sr(a) é tratado(a) com menos respeito do que as outras pessoas.
*3. You receive poorer service than other people at restaurants or stores.*	3. Em restaurantes e lojas, o(a) Sr(a) recebe um atendimento de pior qualidade do que as outras pessoas.
*4. People act as if they think you are not smart.*	4. As pessoas agem como se o(a) Sr(a) não fosse inteligente.
*5. People act as if they are afraid of you.*	5. As pessoas agem como se tivessem medo do(a) Sr(a).
*6. People act as if they think you are dishonest.*	6. As pessoas agem como se o(a) Sr(a) fosse desonesto(a).
*7. People act as if they’re better than you are.*	7. As pessoas agem como se fossem melhores do que o(a) Sr(a).
*8. You are called names or insulted.*	8. O(a) Sr(a) é xingado(a) ou insultado(a)/ofendido(a).
*9. You are threatened or harassed.*	9. O(a) Sr(a) é ameaçado(a) ou assediado(a)/ constrangido(a).
*10. You are followed around in stores.*	10. O(a) Sr(a) é tratado(a) de forma suspeita e é vigiado(a) em lugares como lojas.
*Answer categories: almost everyday, at least once a week, a few times a month, a few times a year, less than once a year, never*	Categorias de resposta: quase todo dia, pelo menos uma vez por semana, algumas vezes por mês, algumas vezes por ano, menos de uma vez ao ano, nunca
*Heightened vigilance scale* (HVS)	Escala de vigilância intensificada (EVI)
*In dealing with these day-to-day experiences that you just told me about, how often do you:*	Vamos continuar pensando em como o(a) Sr(a) lida com aquela(s) situação(ões) que relatou anteriormente... No seu dia a dia, com que frequência o(a) Sr(a)…
*1. Think in advance about the kinds of problems you are likely to experience?*	1. ... se prepara com antecedência para os problemas que poderá enfrentar?
*2. Try to prepare for possible insults before leaving home?*	2. ... tenta se preparar para possíveis xingamentos ou insultos ou ofensas antes de sair de casa?
*3. Feel that you always have to be very careful about your appearance to get good service or avoid being harassed?*	3. ... sente que o(a) Sr(a) sempre tem que ser muito cuidadoso(a) em relação à sua aparência para obter um bom serviço ou para evitar ser assediado(a)?
*4. Carefully watch what you say and how you say it?*	4. ... fica cuidadosamente atento (a) ao que fala e como o(a) Sr(a)fala?
*5. Carefully observe what happens around you?*	5. ... observa com cuidado o que acontece no seu entorno?
*6. Try to avoid certain social situations and places?*	6. ... tenta evitar certos eventos sociais e certos lugares?
*Answer categories: very often, fairly often, not too often, hardly ever, never*	Categorias de resposta: com muita frequência, com frequência, com pouco frequência, quase nunca, nunca

### Estrutura Dimensional e Invariância entre Subgrupos

Os participantes do estudo eram em sua maioria mulheres (56%), na faixa etária de 40 a 59 anos (53,8%), com ensino superior completo (58%) e autorreferidos como brancos (52%). Características semelhantes foram observadas entre os participantes da subamostra do estudo de confiabilidade teste-reteste.

Na AFC para a escala de discriminação (Tabela 1), o modelo inicial, sem correlações entre os itens, apresentou cargas entre 0,527 (item 5) e 0,865 (item 2), com índices de ajuste afastados dos limites recomendados: CFI = 0,931; TLI = 0,911; RMSEA = 0,122. Após a inserção das correlações residuais entre os itens 1 e 2, os índices de ajuste melhoraram. O resultado com fator único mostrou um ajuste razoável com melhoria dos indicadores após uma correlação adicional: entre os itens 8 e 9 da escala: CFI = 0,980; TLI = 0,973; RMSEA = 0,067. A VME (0,402) foi inferior ao limite aceitável (≥ 0,500), mas a CC (0,869) alcançou valor preferível (≥ 0,600). Esse modelo da AFC com duas correlações entre itens, serviu de base para os modelos configurais por subgrupo de características sociodemográficas e as análises de invariância na EDD.

**Tabela 1:** Análise fatorial confirmatória e parâmetros de ajuste para a escala de discriminação no dia a dia (EDD), ELSA-Brasil, 2017–2019, (n = 11.987).

Itens da escala, correlações residuais e índices de ajuste	AFC (sem correlações)		AFC (correlação 1 e 2)		AFC final	
		
	CF	R	CF	R	CF	R
Escala de discriminação no dia a dia						
1. Tratado(a) com menos gentileza	0,825	0,319	0,671	0,550	0,676	0,542
2. Tratado(a) com menos respeito	0,865	0,252	0,724	0,476	0,731	0,466
3. Recebe atendimento de pior qualidade	0,648	0,580	0,673	0,547	0,677	0,542
4. Pessoas agem como se o(a) Sr(a) não fosse inteligente	0,726	0,473	0,757	0,426	0,762	0,420
5. Pessoas agem como se tivessem medo do(a) Sr(a)	0,527	0,723	0,546	0,702	0,549	0,699
6. Pessoas agem como se fossem melhores do que o(a) Sr(a)	0,714	0,492	0,734	0,461	0,740	0,453
7. Pessoas agem como se o(a) Sr(a) fosse desonesto(a)	0,710	0,496	0,750	0,437	0,754	0,431
8. É xingado(a) ou insultado(a)/ofendido(a)	0,678	0,540	0,705	0,503	0,667	0,555
9. É ameaçado(a) ou assediado(a)/constrangido(a)	0,678	0,541	0,701	0,508	0,657	0,568
10. É tratado(a) de forma suspeita e é vigiado(a) em lugares como lojas	0,637	0,594	0,656	0,569	0,661	0,564
Correlação residual – itens 1 e 2	-	-	0,605	-	0,599	-
Correlação residual – itens 8 e 9	-	-	-	-	0,329	-
Índices de ajuste						
Índice de ajuste comparativo e de Tucker-Lewis (CFI/TLI)	0,931/0,911		0,973/0,964		0,980/0,973	
RMSEA (IC95%)	0,122 (0,120–0,125)		0,078 (0,075–0,810)		0,067 (0,065–0,700)	
Variância média extraída (VME)	-		-		0,402	
Confiabilidade composta (CC)	-		-		0,869	

AFC: análise fatorial confirmatória; CF: cargas fatoriais padronizadas; R: resíduos; RMSEA: raiz do erro quadrático médio de aproximação; IC95%: intervalo de confiança de 95%.

Para a avaliação da EVI, procedeu-se à AFE, inicialmente, pelo método de extração dos eixos principais, rotação oblíqua. O índice de KMO para adequação da amostra foi de 0,75, considerado bom, indicando que a matriz era fatorável. O teste de esfericidade de Bartlett com níveis de significância p < 0,00 foi considerado adequado, indicando que a matriz correlacional não era uma matriz identidade. As análises paralelas indicaram a retenção de dois fatores. A análise fatorial exploratória com um fator apresentou cargas muito baixas para os itens 5 e 6: 0,495 e 0,363, respectivamente. Esse modelo configural também não apresentou bons indicadores de ajuste: CFI = 0,918; TLI = 0,864; RMSEA = 0,138. Os resultados da AFE com dois fatores melhoraram esses indicadores, mas apresentou cargas cruzadas nos itens 3 e 6, além de resíduos com valores altos. A AFC com solução de fator único apresentou resultados aceitáveis após inserção de correlações residuais entre os itens 1 e 2 e entre os itens 4 e 5 da escala: CFI = 0,991; TLI = 0,980; RMSEA = 0,053. No entanto, os itens 5 e 6 continuaram com cargas inferiores a 0,500. O resultado da confiabilidade composta para esse modelo (0,668) foi satisfatório, mas a validade convergente (0,258) foi inferior ao recomendado (Tabela 2). Com cargas baixas e resíduos altos na análise fatorial confirmatória da EVI, optamos por não prosseguir com os modelos configurais por subgrupo e com os testes para equivalência de mensuração dessa escala.

**Tabela 2:** Análise fatorial exploratória, análise fatorial confirmatória e parâmetros de ajuste para a escala de vigilância intensificada (EVI), ELSA-Brasil, 2017–2019, (n = 8.916).

Itens da escala, correlações residuais e índices de ajuste	AFE (1 fator)		AFE (2 fatores)			AFC		AFC final	
			
	CF	R	CF1	CF2	R	CF	R	CF	R
**Escala de vigilância intensificada**									
1. Se prepara com antecedência para os problemas que poderá enfrentar	0,698	0,512	0,535	0,215	0,563	0,531	0,718	0,564	0,682
2. Tenta se preparar para possíveis xingamentos ou insultos ou ofensas antes de sair de casa	0,771	0,406	0,991	0,003	0,020	0,596	0,644	0,634	0,599
3. Sente que sempre tem que ser muito cuidadoso(a) em relação à sua aparência	0,577	0,667	0,280	0,382	0,679	0,627	0,606	0,653	0,574
4. Fica cuidadosamente atento(a) ao que fala e como o(a) Sr(a) fala	0,641	0,589	0,002	0,824	0,322	0,716	0,487	0,641	0,589
5. Observa com cuidado o que acontece no seu entorno	0,495	0,755	0,000	0,559	0,688	0,534	0,715	0,434	0,812
6. Tenta evitar certos eventos sociais e certos lugares	0,363	0,868	0,132	0,287	0,866	0,387	0,850	0,395	0,844
Correlação residual – itens 1 e 2	-	-	-	-	-	0,456	-	0,422	-
Correlação residual – itens 4 e 5	-	-	-	-	-	-	-	0,265	-
**Índices de ajuste**									
Índice de ajuste comparativo e de Tucker-Lewis (CFI/TLI)	0,918 / 0,864		0,996 / 0,986			0,973 / 0,949		0,991 / 0,980	
RMSEA (IC95%)	0,138 (0,132–0,144)		0,044 (0,035–0,053)			0,085 (0,078–0,091)		0,053 (0,046–0,060)	
Correlação das dimensões	-		0,456			-		-	
Variância média extraída (VME)	-		-			-		0,258	
Confiabilidade composta (CC)	-		-			-		0,668	

AFE: análise fatorial exploratória; AFC: análise fatorial confirmatória; CF: cargas fatoriais padronizadas; R: resíduos; RMSEA: raiz do erro quadrático médio de aproximação; IC95%: intervalo de confiança de 95%.

A avaliação da equivalência de mensuração para a EDD mostrou indicadores CFI (≥ 0,95) e RMSEA (≤ 0,080) com ajustes aceitáveis para invariância configural nas comparações dos quatro grupos. Na invariância métrica, esses indicadores de ajuste melhoraram em todas as comparações de grupos, ou seja, o RMSEA foi reduzido significativamente, sem sobreposição do IC95% com a estimativa do modelo anterior, e houve incremento no CFI que passou a ser superior a 0,990 em todos os casos. O modelo da invariância escalar indicou constância apenas para comparações nos subgrupos de sexo. Para comparação entre faixas etárias, a redução no CFI foi limítrofe passando de 0,992 no modelo de invariância métrica para 0,981 no modelo de escalar (Δ de -0,011), enquanto o aumento no RMSEA foi tolerável (Δ de 0,007). A invariância escalar não foi alcançada para comparações nos subgrupos de raça/cor da pele e de escolaridade. Em ambos os casos o incremento no RMSEA foi superior a 0,015 (0,023 e 0,016, respectivamente) e a redução no CFI foi maior que 0,010 (-0,024 e -0,018, respectivamente) (Tabela 3).

**Tabela 3:** Comparação da invariância entre subgrupos de características socioeconômicas para equivalências configural, métrica e escalar da escala de discriminação no dia a dia. ELSA-Brasil, 2017–2019 (n = 11.987).

Equivalência de mensuração	Parâmetros e índices de qualidade do ajuste dos modelos							Comparação de modelos (p)	
	
	χ^2^ (gl)	p	Parâmetros livres	RMSEA (IC95%)	∆_RMSEA_	CFI	∆_CFI_	Métrica e configural	Escalar e métrica
Raça/cor da pele									
Invariância configural	1.731,539 (66)	< 0,0001	124	0,066 (0,064–0,069)	0,001	0,981	0,001	-	-
Invariância métrica	731,981 (76)	< 0,0001	114	0,039 (0,036–0,041)	-0,027	0,993	0,012	0,0348	-
Invariância escalar	2.878,628 (126)	< 0,0001	64	0,062 (0,060–0,064)	0,023	0,969	-0,024	-	< 0,0001
Sexo									
Invariância configural	1.875,612 (66)	< 0,0001	124	0,068 (0,065–0,070)	0,003	0,980	0,000	-	-
Invariância métrica	876,957 (76)	< 0,0001	114	0,042 (0,039–0,044)	-0,026	0,991	0,011	< 0,0001	-
Invariância escalar	1.052,410 (126)	< 0,0001	64	0,035 (0,033–0,037)	-0,007	0,990	-0,001	-	< 0,0001
Faixas etárias									
Invariância configural	1.911,151 (66)	< 0,0001	124	0,068 (0,066–0,071)	0,003	0,980	0,000	-	-
Invariância métrica	825,650 (76)	< 0,0001	114	0,041 (0,038–0,043)	-0,027	0,992	0,012	0,0073	-
Invariância escalar	1.876,840 (126)	< 0,0001	64	0,048 (0,046–0,050)	0,007	0,981	-0,011	-	< 0,0001
Escolaridade									
Invariância configural	1.856,748 (66)	< 0,0001	124	0,068 (0,065–0,070)	0,003	0,981	0,001	-	-
Invariância métrica	855,991 (65)	< 0,0001	114	0,042 (0,039–0,044)	-0,026	0,992	0,011	< 0,0001	-
Invariância escalar	2.604,448 (126)	< 0,0001	64	0,058 (0,056–0,060)	0,016	0,974	-0,018	-	< 0,0001

gl: graus de liberdade; RMSEA: raiz do erro quadrático médio de aproximação; IC95%: intervalo de confiança de 95%; CFI: índice de ajuste comparativo.

### Estudo de Confiabilidade Teste-reteste

Para a EDD, os escores no teste e no reteste variaram respectivamente de 10 a 48 (média = 18,07) e de 10 a 51 (média = 17,05). Para a EVI, variou de sete a 29 (média = 19,78) no teste e de sete a 30 no reteste (média = 18,92). O Kp na EDD variou de 0,39 (item 9) a 0,78 (item 10), para a EVI variou de 0,47 (item 3) e 0,56 (item 4). Os CCI variaram de 0,87 (IC95% 0,83–0,90) para EDD e 0,83 (IC95% 0,76–0,88) para EVI (Tabela 4).

**Tabela 4:** Confiabilidade teste-reteste da escala de discriminação no dia a dia (EDD) e da escala de vigilância intensificada (EVI), ELSA-Brasil, 2017–2019.

	kappa ponderado (IC95%)	Coeficiente de correlação intraclasse
Escala de discriminação no dia a dia (n = 260)		
1. Tratado(a) com menos gentileza	0,59 (0,49–0,69)	0,87 (IC95% 0,83–0,90)
2. Tratado(a) com menos respeito	0,58 (0,48–0,68)	
3. Recebe atendimento de pior qualidade	0,58 (0,45–0,72)	
4. Pessoas agem como se o(a) Sr(a) não fosse inteligente	0,66 (0,56–0,76)	
5. Pessoas agem como se tivessem medo do(a) Sr(a)	0,63 (0,51–0,76)	
6. Pessoas agem como se fossem melhores do que o(a) Sr(a)	0,65 (0,48–0,81)	
7. Pessoas agem como se o(a) Sr(a) fosse desonesto(a)	0,62 (0,52–0,71)	
8. É xingado(a) ou insultado(a)/ofendido(a)	0,46 (0,32–0,60)	
9. É ameaçado(a) ou assediado(a)/constrangido(a)	0,39 (0,20–0,57)	
10. É tratado(a) de forma suspeita e é vigiado(a) em lugares como lojas	0,78 (0,68–0,88)	
Escala de vigilância intensificada (n = 149)		
1. Se prepara com antecedência para os problemas que poderá enfrentar	0,50 (0,36–0,63)	0,83 (IC95% 0,76–0,88)
2. Tenta se preparar para possíveis xingamentos ou insultos ou ofensas antes de sair de casa	0,51 (0,37–0,65)	
3. Sente que sempre tem que ser muito cuidadoso(a) em relação à sua aparência	0,47 (0,32–0,61)	
4. Fica cuidadosamente atento(a) ao que fala e como o(a) Sr(a) fala	0,56 (0,42–0,71)	
5. Observa com cuidado o que acontece no seu entorno	0,53 (0,37–0,70)	
6. Tenta evitar certos eventos sociais e certos lugares	0,51 (0,38–0,65)	

IC95%: intervalo de confiança de 95%.

## DISCUSSÃO

Seguindo as etapas recomendadas pela literatura^20^ sobre adaptação transcultural, os resultados mostraram que a versão brasileira da EDD apresenta adaptação transcultural aceitável, o que permite seu uso futuro em estudos epidemiológicos. No entanto, nossas análises não corroboram o uso da EVI relacionada à discriminação no formato atual.

Em relação à EDD, nossa análise apoiou a unidimensionalidade da escala, semelhante a outros estudos^2,22^, e foi consistente com pesquisas anteriores sobre dependência local ou alta correlação entre os itens 1 e 2 e entre os itens 8 e 9^13,22,28^. Estudo psicométrico que também explorou qualitativamente a escala indicou que a correlação entre os itens 1 e 2 pode ser devida à redundância, uma vez que esses itens foram vistos com significado semelhante pelos entrevistados^28^, embora isso não tenha sido captado nos pré-testes realizados no âmbito do ELSA-Brasil. Já a correlação entre os itens 8 e 9 pode ser explicada pela natureza dessas experiências, relacionadas a formas agudas de discriminação, diretamente endereçadas e mais evidentes, e se distanciando da natureza sutil ou crônica das experiências para as quais a EDD foi proposta^22^.

Levando em conta essas avaliações, uma versão resumida da escala de discriminação com cinco itens (α = 0,77) foi desenvolvida para o *Chicago Community Adult Health Study*^29^ e contempla mudanças em itens que identificamos como correlacionados: os dois primeiros itens da escala foram unidos (*treated with less courtesy/respect than other people*) e o item 8 foi retirado, mantendo-se apenas o item 9, acrescido do termo “constrangido”. Não foram incluídos na versão resumida os itens 6, 7 e 10. Incentivamos, portanto, que pesquisas futuras no Brasil e outros países de língua portuguesa avaliem a adequação de versões resumidas modificadas da escala.

Os modelos utilizados para avaliar a equivalência de mensuração entre grupos na escala de discriminação indicaram invariância métrica em todos os casos, fornecendo evidência de que os respondentes usam a escala de maneira semelhante entre os subgrupos, então as diferenças entre valores podem ser comparadas^24^. No entanto, a invariância escalar não foi alcançada para comparações entre grupos raciais e de diferentes níveis de escolaridade. Esses resultados confirmam as conclusões de estudos mais recentes sobre invariância de medição na EDD, que relataram, ao considerar a discriminação geral sem atribuir motivação, falta de equivalência (ou não invariância) entre grupos raciais e com base na escolaridade^12,13^. Esses estudos, entretanto, divergiram em relação à invariância entre demais subgrupos: enquanto um indicou que apenas comparações entre grupos etários das estimativas da escala de discriminação podem ser consideradas significativas^13^, o outro sugeriu que comparações entre homens e mulheres são adequadas^12^. Ressalta-se, no entanto, que a mudança nos valores de CFI adotada nesses estudos, com reduções ≥ 0,002, foram consideradas uma indicação de não invariância entre os grupos. Esse ponto de corte foi mais conservador do que aquele adotado em nossas análises.

Em relação à EVI, observou-se indicadores não aceitáveis e, até onde foi possível avaliar, poucos estudos avaliaram as propriedades psicométricas dessa escala, o que limita comparações. Desafios semelhantes relacionados à dimensionalidade da escala têm sido relatados em outros estudos. Por exemplo, um estudo nos EUA indicou duas dimensões nas análises fatoriais exploratória e confirmatória: uma relacionada ao que os autores denominaram *preparation* (do item 1 ao 3) e outra relacionada ao que denominaram de *caution* (do item 4 ao 6)^19^. O mesmo estudo, ao utilizar um modelo de fator único, encontrou ajuste insatisfatório (CFI = 0,94 e RMSEA = 0,12) e optaram por seguir as análises com modelo de dois fatores com melhoria nos indicadores (CFI = 0,98 e RMSEA = 0,07). Informação adicional sobre a estrutura da escala vem do estudo que publicou originalmente a EVI e reportou análise por componentes principais, com um componente (autovalor = 3,42) responsável por 57% da variância padronizada (cargas > 0,69)^15^.

Esses aspectos têm motivado também a revisão dessa escala e uma versão resumida com quatro itens (α = 0,72) foi aplicada no *Chicago Community Adult Health Study*^30^. Essa versão retira dois itens que estavam envolvidos em correlações residuais em nossa análise: os itens 1 e 5. Outro estudo utilizou uma versão ainda mais resumida da EVI com retirada também do item 4 (α = 0,66)^16^ e LaVeist et al.^17^ usaram a versão original, com a retirada do item 6 (α = 0,69).

Embora a EVI de forma abreviada seja utilizada em pesquisas sobre sua associação com desfechos de saúde, avaliações psicométricas adicionais são necessárias em contextos distintos para elucidar se a escala pode ser utilizada na população e na comparação entre subgrupos. Encontramos apenas um estudo que avaliou a invariância de medição da EVI, no qual comparações entre transgênero e cisgênero e entre subgrupos de cisgênero puderam ser realizadas, pois a escala funciona de forma equivalente entre esses grupos. Por outro lado, as comparações entre subgrupos de transgêneros exigiram cautela, uma vez que foi encontrada invariância métrica parcial e invariância escalar parcial^19^.

O presente estudo apresenta como pontos fortes a qualidade do processo de obtenção dos dados, além de uma apreciação bastante ampla das avaliações que compõem as etapas de adaptação transcultural em uma amostra abrangente no país e com diferentes características sociodemográficas. No entanto, a composição da população do ELSA-Brasil – adultos e idosos, empregados ou aposentados, e com escolaridade mais elevada do que a média da população brasileira – limitam a sua representatividade.

Por fim, destacamos que a EDD obteve resultados psicométricos aceitáveis para utilização no ELSA-Brasil e em populações similares. Não foi possível identificar propriedades psicométricas aceitáveis para a escala de vigilância. No entanto, dada a importância do tema em estudos epidemiológicos na realidade brasileira, aconselha-se que as versões resumidas propostas mais recentemente sejam utilizadas em novos estudos e avaliadas sobre sua pertinência no nosso contexto.
